# Effects of prenatal exposure to diesel exhaust particles on postnatal development, behavior, genotoxicity and inflammation in mice

**DOI:** 10.1186/1743-8977-5-3

**Published:** 2008-03-11

**Authors:** Karin S Hougaard, Keld A Jensen, Pernille Nordly, Camilla Taxvig, Ulla Vogel, Anne T Saber, Håkan Wallin

**Affiliations:** 1 National Research Centre of the Working Environment, Lersø Parkallé 105, DK-2100 Copenhagen Ø, Denmark; 2 Faculty of Pharmaceutical Sciences, University of Copenhagen, Universitetsparken 2, DK-2100 Copenhagen Ø, Denmark; 3 National Food Institute, Technical University of Denmark, Mørkhøj Bygade 19, DK-2860 Mørkhøj, Denmark

## Abstract

**Background:**

Results from epidemiological studies indicate that particulate air pollution constitutes a hazard for human health. Recent studies suggest that diesel exhaust possesses endocrine activity and therefore may affect reproductive outcome. This study in mice aimed to investigate whether exposure to diesel exhaust particles (DEP; NIST 2975) would affect gestation, postnatal development, activity, learning and memory, and biomarkers of transplacental toxicity. Pregnant mice (C57BL/6; BomTac) were exposed to 19 mg/m^3 ^DEP (~1·10^6 ^particles/cm^3^; mass median diameter ≅ 240 nm) on gestational days 9–19, for 1 h/day.

**Results:**

Gestational parameters were similar in control and diesel groups. Shortly after birth, body weights of DEP offspring were slightly lower than in controls. This difference increased during lactation, so by weaning the DEP exposed offspring weighed significantly less than the control progeny. Only slight effects of exposure were observed on cognitive function in female DEP offspring and on biomarkers of exposure to particles or genotoxic substances.

**Conclusion:**

In utero exposure to DEP decreased weight gain during lactation. Cognitive function and levels of biomarkers of exposure to particles or to genotoxic substances were generally similar in exposed and control offspring. The particle size and chemical composition of the DEP and differences in exposure methods (fresh, whole exhaust versus aged, resuspended DEP) may play a significant role on the biological effects observed in this compared to other studies.

## Background

Results from epidemiological studies indicate that particulate air pollution constitutes a hazard for human health, by increasing the prevalence of e.g., cardiovascular and lung disease [1]. Air pollution has also been associated with adverse reproductive outcomes in humans, although further studies are needed for most endpoints to confirm causal relationships and the role of different air pollutant sources [2].

Exhaust from diesel engines is thought to be an important component of air pollution and diesel exhaust particles (DEP) contribute significantly to the particulate air pollution in urban air [3]. Reproductive function effects of diesel exhaust have been assessed in controlled animal studies (Table 1). Malforming properties have not been revealed [4-7], but functional domains may be affected by prenatal exposure to diesel exhaust. Thus, prenatal exposure to diesel exhaust has been associated with changes in e.g. offspring body weight [5,8] and immunological measures [9], and in mice transplacental exposure to diesel exhaust particles increased the frequency of DNA deletions in fetal tissue [10]. In vivo data also indicate that diesel exhaust possesses endocrine activity, i.e. prenatal exposure to diesel exhaust affects sexual differentiation and function [4,7-9,11]. Exposure of growing male rats to diesel exhaust has been associated with changes in reproductive endocrine function and reduced sperm production and activity [12,13]. All studies used whole diesel exhaust, i.e. exposure to exhaust gases in combination with DEP, although some studies also used filtered exhaust, where the exposure atmosphere contained only gaseous exhaust and particles measuring less than 0.05–0.2 μm. A numerically large fraction of the particulates in diesel exhaust is ultra fine in size [3] and recent studies indicate that the particulate fraction may be very important for toxicity [14,15]. The primary aim of the present study was to determine whether prenatal exposure to resuspended DEP affected reproductive parameters, postnatal development and behavior, and secondly whether DEP would affect biomarkers of inflammation and DNA damage in the offspring.

**Table 1:** Overview of animal studies with maternal inhalation exposure to whole diesel exhaust

*Diesel particulate in air (mg/m^3^)*	*Particle size*	*Study type*	*Animal species*	*Exposure period (GD)*	*Maternal measures (n)*	*Offspring endpoints (n)*	*Ref.*
0.3 1.0 3.0	MMAD 0.4	T(13)	M	2–13 12 h/day	(23–30) Implantations Litter size Sex ratio Placentas: -weight ^[↑1.0]^ -congestion -abnormal -inflammatory cytokines ^[↑0.3, 3.0]^	Fetal weight ^[↓3.0]^ Rate of abnormal fetuses	[5]
0.3 1.0 3.0	MMAD 0.4	P	M	From four months before gestation to mating 12 h/day	(10–12) Abnormal delivery Nest construction ^[↓all]^ Weights: -ovaries -vagina -uterus ^[↓1.0]^	(4–22) Weight, PND11 ^[♂↑0.3]^, 14, 21 ^[♀↓3.0]^, 28 ^[↓3.0]^, 35 ^[↓3.0]^, 42 ^[↓0.1, 3.0]^, 49 ^[↓0.1♂, 3.0]^, 56 ^[↓1.0, 3.0]^, 63 ^[↓1.0♀, 3.0]^, 70 ^[↓3.0]^ Vaginal opening ^[↓0.3, 1.0]^ Anogenital distance PND28 ^[♂↓0.3]^, 70 ^[♂↓3.0]^ ♀ Weights, PND28 -thymus ^[↓3.0]^ -ovary ^[↓3.0]^ ♀ Weights, PND70 -adrenals ^[↓1.0]^ -liver ^[↓1.0]^ -thymus ^[↓0.3, 1.0]^ ♂ Weights, PND 70 -adrenals ^[↓1.0]^ -testes ^[↓1.0]^ -seminal vesicles ^[↓1.0]^ External malformations	[4]
0.1		T(13)	M	2-3+6-10+13 8 h/day	(15–16) Body weight Implantations Total placental weight	(22) Litter size Gender ratio Total fetal weight Bone morphogenetic protein 15^[↓]^	[20]
1.71 or 0.17 (Filtered, pore size estimated to 0.1–0.2 μm)		P	R	7–20 6 h/day	(7–8)	(7–14) Gender ratio Weight, PND 96: -body -testis -epididymis Daily sperm production ^[↓]^ Spermatids ^[↓]^ Sertoli cells ^[↓]^ Spermatids/Sertoli cell ^[↓]^ FSH ^[↓]^ LH Testosterone	[11]
5.6 or Filtered (pore size 0.05 μm)	90% < 0.5 μm	T(20)	R	7–20 6 h/day	(22–24) Body weight Implantations Weights: -placenta ^[↓, non-filtered]^ -adrenal gland ^[↓, filtered]^ -thymus ^[↓]^ -ovary ^[↓]^ Corpora lutea ^[smaller]^ Testosterone ^[↑]^ Progesterone Estradiol ^[↓, filtered]^ LH ^[↓, filtered]^ FSH ACTH Hydroxycorticosteroids, urinary	(6–24) Fetal weight ^[females↑]^ Litter size Gender ratio External malformations Anogenital distance ^[↑]^ Thymus weight ^[↓]^ Testis differentiation ^[↓]^ Ovary differentation ^[↓]^ Thymus differentiation ^[↓]^	[7]
1.73 or Filtered (pore size 0.05 μm)	90% < 0.5 μm	P	R	7-20+ PND 1–3 6 h/day	(6–7)	(6–20, males only) Weight PND 4, 49 Organ weights, PND 4, 49, 82: -thymus ^[↓]^ -Testosterone, PND 4, 23 ^[↑]^ -Estrogen, PND 4, 23 IgE pollen: -three immunizations -four immunizations ^[↑]^	[9]
6	~90% < 1 μ ~50% < 0.3 μ	T(20)	R	5–16 8 h/day	Resorptions Implantations Corpora luetea ^[↓]^ Gross pathology	Fetal weight Litter size Viability Gender distribution Malformations	[6]
6	~90% < 1 μ ~50% < 0.3 μ	T(20)	Rabbit	5–16 8 h/day	(20) Resorptions Implantations Corpora lutea Gross pathology	Fetal weight Litter size Viability Gender distribution Malformations	[6]
0.3 1.0 3.0		P	M	2–16 12 h/day	(20) Deaths	(7–14, males only) Weight, PND 28 ^[↑1.0, 3.0]^ Gender ratio Organ weights, PND 28 -testes ^[↑1.0, 3.0]^ -epididymis -accessory glands ^[↑1.0, 3.0]^ LH Testosterone ^[↑1.0]^	[8]
1.0		P	M	2–16 24 h/day	(10)	(7–10) Weight, PND 8 ^[↓]^, 16^(↓)^, 21^(↓)^, 35 ^[↓]^, 84: -testis -epididymis -seminal vesicle ^[↑, PND8+16]^ Sperm counts ^[↓PND35+84]^ Testis morphology ^[↑Multinucl. cells PND84]^ Testosterone ^[↓PND21,(↓)PND35, ↑PND84]^ mRNA, testes: -androgen receptor -estrogen receptor -LH receptor -FSH receptor -steroidogen acute regul protein ^[↑PND84]^ -cytochrome P-450 side-chain cleavage -3βHSD -17βHSD -P450c17 -cytochrome P-450 aromatase	[19]

n: group size. GD: Gestation day. PND: Postnatal day. Study type: P: Postnatal assessment of effects. T(GD): Teratology study, number in brackets designates day of laparatomy. Species: M: Mice. R: Rat. [Square brackets]: indication of dose levels and gender related to exposure associated effect. ↓: decrease; ↑: increase. MMAD: Mass median aerodynamic diameter. LH: Luteinizing hormone. FSH: Follicle stimulating hormone. ACTH: Adrenocorticotropic hormone. HSD: Hydroxysteroid dehydrogenase.

## Results

### Exposure evaluation

The inhalation exposure was performed on a daily basis on 13 successive days for both DEP and control air, each organized in two groups. The DEP mass concentration was controlled by filter sampling and showed the mean mass concentration in the exposure chamber was 19.1 ± 1.13 (SEM) mg/m^3^DEP.

**Figure 1:**
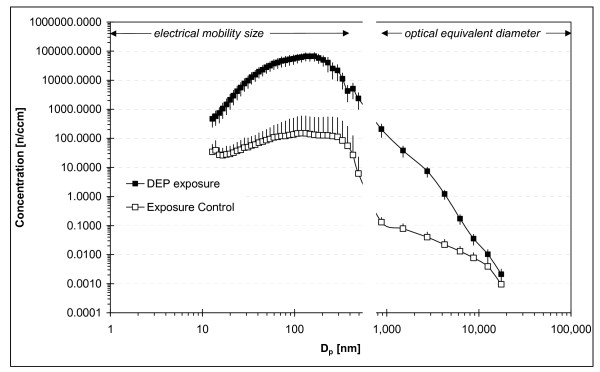
**Average particle number distribution during DEP-exposure compared to the average particle size distribution in exposure to controls.***n *= 26. Error bars denote the SD of the average concentrations calculated based on the average for each N exposure.

The resuspended DEP showed maximum concentrations (66,374 ± 21,159 (SD) n/cm^3^) for 132 to 163.5-nm-size particles (geometric mean = 146.9 nm) and the total concentration exceeded ~9.86·10^5 ^± 0.08·10^5 ^(SD) particles/cm^3 ^(Figure 1). The particle size distribution for the aerosolized DEP for both exposure groups was identical allowing us to treat the groups as one exposure group. A similar conclusion was reached for the control group. The control group was exposed to lab air diluted by HEPA filtered compressed air and minor concentrations of re-aerosolized DEP particles (3.07·10^3 ^± 0.16·10^3 ^(SD) particles/cm^3^) deposited onto inner surfaces in the exposure system. This was considered clean air and the exposure levels to the control groups were assumed negligible. Similar methodology has been applied in several previous studies with no biological effect observed.

**Figure 2:**
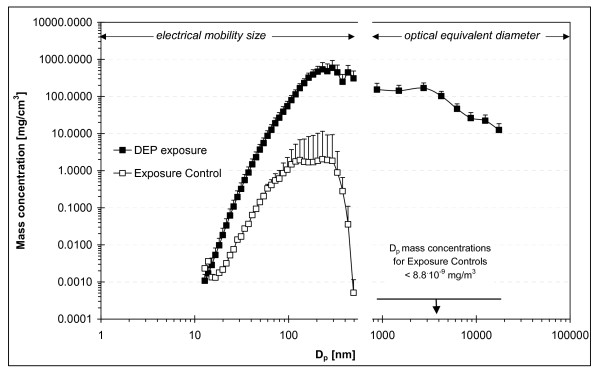
**Average mass number distribution during DEP-exposure compared to the average mass distribution in exposure to controls.***n *= 26. Error bars denote the SD of the average concentrations calculated based on the average for each N exposure.

Calculated in mass number concentrations, the peak concentration appeared at 292 nm (Figure 2) and the mass median diameter of the airborne DEP was 240 nm (upper and lower quartile of 182 and 353 nm, respectively. The true mass median diameter may be slightly higher as the coarsest particle sizes were not determined. Assuming the same density for all particle size-fractions, less than 10 wt% of the aerosolized DEP consisted of particles coarser than 1 μm, where gastro-intestinal uptake becomes important) [16]. Bronchial and pulmonary deposition in mice is high for fine particles, reaching 14 and 45 wt% at 270 nm, respectively [16]. We were unable to locate data in the literature on deposition efficiencies in mice for particles below 270 nm. A conservative estimate of the deposition efficiencies based on polynomial extrapolation from Raabe et al. suggests that ~70 wt% of the total inhaled airborne DEP was deposited in the pulmonary and alveolar space of the mice [16]. These data further suggest that ~10 wt% of the inhaled DEP would be deposited in the gastro-intestinal tract (GI) within 24 hours after the exposure) [16]. However, the mice may have received a higher dose to the GI after exposure owing to their cleaning of the fur.

### Maternal measures

No clinical signs of toxicity were observed in the dams during the exposure period and the number of females presenting offspring was similar in the two groups. DEP females tended to gain more weight during gestation than did controls [DEP weight gain 14.73 ± 0.59 g (SEM) vs. weight gain in controls: 12.98 ± 0.66 g (SEM), *p *= 0.05], but lactational body weights and absolute and relative maternal organ weights at weaning were similar in control and DEP dams.

### Litter parameters

**Figure 3:**
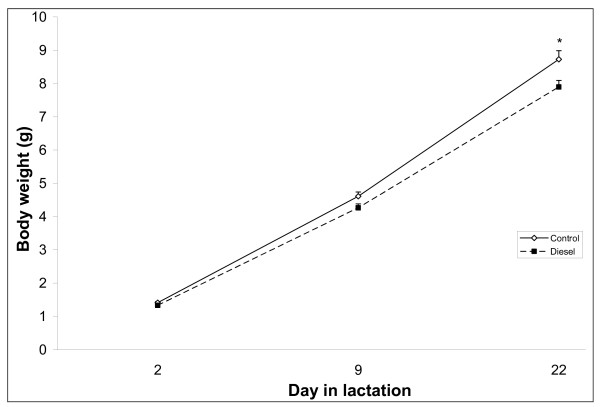
**Average litter weight during lactation.** Litters were either controls or exposed to DEP during gestation. Data represent mean+SEM. Mean Offspring exposed to DEP during fetal life gained statistically significantly less weight during the latter half of lactation compared to control offspring. *n *= 12. *p < 0.05.

Litter size, gender ratio, and implantation loss did not differ between groups, but statistically lactational body weights were significantly lower in DEP than in control offspring (Figure 3). Post hoc analyses showed that shortly after birth, body weights of DEP offspring were numerically, but not statistically significantly lower than in controls [controls 1.41 ± 0.03 g (SEM) vs. DEP 1.33 ± 0.04 g (SEM)]. However, by weaning DEP offspring weighed approximately 10% less than control progeny. When weight gain from postnatal day (PND) 2 to 9 and from PND 9 to 22 was analyzed, the overall analysis showed weight gain to be statistically significantly lower in DEP litters compared to control litters. Post hoc analysis indicated that the decreased weight gain was only statistically different in the latter part of gestation. At weaning, one male and one female from each litter were selected for behavioral testing. Body weights of these randomly selected animals did not differ at the day of weaning, and when the weights of these animals were analyzed until termination of behavioral tests, no differences pertaining to prenatal exposure were revealed.

### Inflammatory and genotoxic endpoints

**Figure 4:**
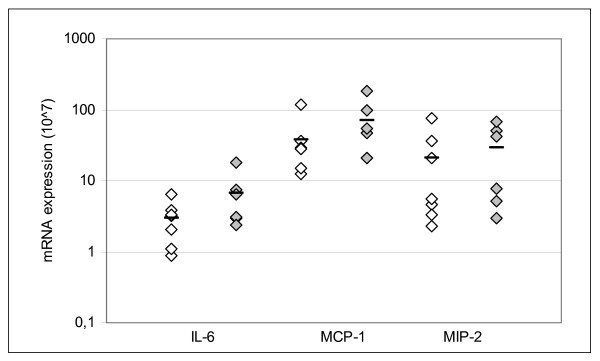
**mRNA expression of inflammatory cytokines in liver from pups at PND 2.** Expression of mRNA is normalized to 18S rRNA. Empty squares: Controls; filled squares: DEP. Horisontal line: mean value. Levels of mRNA of IL-6, MCP-1, and MIP-2 were normalized to the 18S levels. *n *= 6–7.

Inflammatory and genotoxic effects of the DEP exposure were evaluated by analysis of gene expression of cytokines and DNA damage in the liver of the offspring. Single cell gel electrophoresis, i.e. the Comet assay, was used to evaluate DNA damage. The tail length of liver cells was similar in controls and the transplacentally DEP exposed offspring [Control 45.0 ± 2.3 (SEM) vs. DEP 50.7 ± 5.3 (SEM)]. DNA damage was also evaluated by analyzing the mRNA expression levels of 8-oxoguanine DNA-glycosylase 1 (OGG1) and excision repair cross complementing group 1 (ERCC1), which are genes involved in DNA repair, as well as heme oxygenase-1 (HO-1), which is expressed during oxidative stress. There was no difference in the mRNA expression of these genes involved in genotoxicity [OGG1: Control 41.01 ± 2.36 (SEM) vs. DEP 41.15 ± 4.72 (SEM); ERCC1: Control 410.8 ± 51.1 (SEM) vs. DEP 437.0 ± 37.7 (SEM); HO-1: Control 3352 ± 426 (SEM) vs. DEP 3219 ± 235 (SEM); mRNA was normalized to 18s rRNA and multiplied by 10^7^]. Thus, there was no indication of DNA damage in the liver of offspring exposed to DEP during fetal life. Inflammation was evaluated by analysis of mRNA expression of the inflammatory cytokines interleukin-6 (IL-6), monocyte chemoattractant protein 1 (MCP-1), and macrophage inflammatory protein 2 (MIP-2). No statistically significant differences were observed between the DEP and air exposed offspring (Figure 4), but the mRNA expression levels were slightly higher in the DEP exposed pups. Thus, there could be a tendency of increased inflammation in the liver of pups from the DEP exposed dams.

### Thyroid hormone analysis

**Figure 5:**
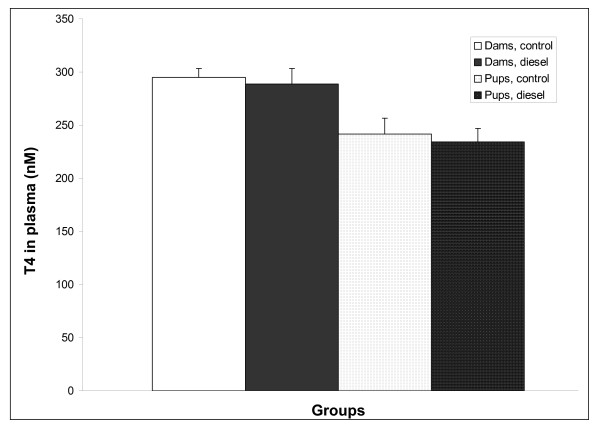
**T4 plasma hormone levels in mothers and offspring.** Blood for T4 plasma hormone levels were collected at the time of weaning and measured by immunoassay. Data represent mean+SEM, *n *= 11–12 for the dams. Data for male and female pups were similar and were therefore collapsed within each prenatal exposure group, *n *= 9.

At weaning, total thyroxin levels (T_4_) in plasma were similar in control and DEP exposed dams and offspring (Figure 5).

### Behavioral tests

**Figure 6:**
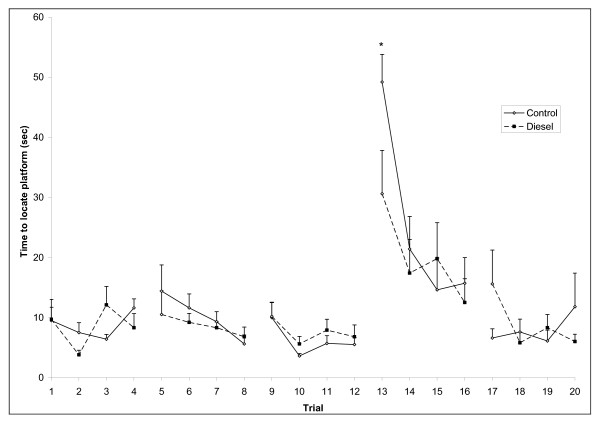
**Memory in the Morris water maze for female offspring.** Data represent mean+SEM. Latency to locate the hidden platform is presented for memory (three days, i.e. trial 1–12; reversal, trial 13–16, and new, trial 17–20). DEP exposed offspring located the platform faster than controls on the first trial of reversal learning. *n *= 10. *p < 0.05.

At the age of two months, male and female offspring were tested for learning and memory ability in the Morris water maze. The mean escape latency for both groups decreased to approximately 10 sec over the course of the 20 learning trials. When memory was tested three weeks later, all animals recalled the location in the south west quadrant, i.e. only a single male animal used the maximally allowed period of 60 sec without climbing onto the platform. When the platform was moved to a new location in the north east quadrant, time to locate the platform increased momentarily and then quickly decreased again. With the platform in the central position the animals quickly learned the position. Both learning and memory performance was similar in the two prenatal exposure groups of both males and females, but female DEP offspring used less time than controls to locate the platform on the first trial of the spatial reversal learning task (*p *< 0.05, Figure 6). A similar, although statistically insignificant, pattern was observed for male DEP offspring (data not shown).

In the Open field, there was a tendency towards increased activity in female DEP offspring [Control 9.77 ± 1.0 m (SEM) vs. DEP 13.5 ± 2.1 m (SEM), (*p *< 0.1)], whereas male offspring from the two groups behaved similarly [Control 14.9 ± 2.4 m (SEM) vs. DEP 15.5 ± 2.5 m (SEM)].

## Discussion

The present study assessed the effects of prenatal exposure to resuspended diesel exhaust particles on gestation, postnatal development, behavior, and biomarkers of prenatal exposure to particles and genotoxic substances. Cognitive function and selected biomarkers only differed slightly between prenatal exposure groups, whereas body weight gain differed between offspring from control and DEP exposed dams, particularly during the latter half of the nursing period. Thus, statistically body weight of DEP pups differed significantly from controls on the day of weaning and rate of weight gain was significantly reduced during the latter half of lactation, i.e. from PND 9 to PND 22.

Growth restriction is a common effect of chemical exposure, also when exposure occurs during fetal life. Thus, chemical exposure of the mother during gestation may be associated with prenatal growth restriction and thus reduced birth weight. When maternal exposure is discontinued at birth, group differences in birth weight may fade with time [17,18]. In the present study, body weights of DEP offspring were slightly, but insignificantly, lower than weights of controls a few days after birth. This difference increased during lactation and body weight was especially affected during the latter part of the period, even if DEP exposure was terminated just prior to birth.

Other studies report effects of diesel exposure during perinatal life on perinatal growth, all using direct exposure to newly generated diesel exhaust (Table 1). Ono et al. observed statistically significantly decreased weight of offspring from ICR mice exposed continuously to whole diesel engine exhaust with 1.0 mg/m^3 ^DEP during gestation day (GD) 2–16, compared to controls at PND 8 and 35 (body weights lower in DEP offspring by 16.4% and 9.5%, respectively). Weights were also lower at GD 16 (by 14.6%) and 21 (by 22.6%), although not significantly so [19]. Since body weights were not recorded prior to PND 8, it is not possible to assess whether diesel exhaust exposure lowered offspring body weights already from birth. In another study, female C57B1 mice were exposed to diesel engine exhaust with 0.3, 1.0, or 3.0 mg/m^3 ^DEP for 12 h/day for four months, terminated at initiation of mating. At the lowest dose levels, the body weights were increased in male diesel pups at PND 11 compared to controls, but no differences were observed later in life for this group. In the highest dose-group body weights were decreased from postnatal week 3 to 10, whereas the middle dose was associated with decreased body weights during postnatal week 6 to 9 [4]. Male offspring from pregnant ICR mice exposed to diesel exhaust with 1.0 or 3.0 mg/m^3 ^DEP 12 h/day during GD 2–16 exhibited increased body weights on PND 28, the only time point measured, but no effect was observed at 0.3 mg/m^3 ^(no data for female offspring). Several studies have measured fetal weights in late gestation after prenatal exposure to whole diesel exhaust, and observed decreased [5], increased (females only [7]) or no effect [6,20] on fetal weight in mice, rabbits or mice.

The report from Tsukue et al. [4] documents effects in the offspring even if exposure to diesel engine exhaust took place during the period preceding mating, indicating that exposure to diesel exhaust do not need to take place concurrently with fetal development for effects to appear [4]. This is possibly owing to gradual release of chemicals attached to diesel particles deposited in maternal lungs, extending the period of internal exposure and thus of induction of effects beyond the period of dosing. Furthermore, substances may be secreted into milk. Pregnant rats were exposed to whole diesel exhaust on GD 7–20, or during the two last weeks of prenatal and two first weeks of postnatal life. Polycyclic aromatic hydrocarbons (PAHs) were measured in fetuses and maternal blood at GD 20, and in breast milk at PND 14. Exposure increased PAH levels in fetal tissues and maternal milk, indicating that PAHs from particles inhaled by the mother transfer to the offspring via the placenta and breast milk [21]. If the effects of DEP are due to soluble compounds leaching from these particles, the blood levels of these compounds may even increase very slowly, and thus the biological effect may be delayed compared to the period of exposure [6]. Finally, chemical substances transferred from mother to fetus may act as toxicants later in life, even if diesel particles were inhaled only during gestation ("programming") [22,23].

Some chemical compounds have been shown to reduce levels of circulating thyroid hormones in developing animals. As a consequence postnatal growth diminishes, because thyroid hormone is a prerequisite for normal growth and development [24,25]. This is for example the case for some polyhalogenated aromatic hydrocarbons [26,27]. It was therefore possible, that toxic agents from the diesel particles had been secreted into maternal milk and ingested by the offspring, where the compounds interfered with the pituitary-thyroid axis and thereby growth, or prenatally transferred substances acted as thyrotoxicants during postnatal life [11]. To pursue this hypothesis, thyroxine (T_4_), the dominating thyroid hormone, was measured in plasma from both dams and offspring. However, T_4 _was similar in exposed and control animals at weaning, the time of maximum observed difference in body weights between control and diesel litters. Thyroid disruption does therefore not seem to be involved in the postnatal growth restriction observed in the present study.

Some animal studies indicate that perinatal exposure to diesel exhaust restricts postnatal growth, whereas other studies report the opposite. Diesel exhaust is produced by the combustion of diesel fuel, which yields a complex mixture of compounds. Therefore the composition varies with fuel type, engine model and tuning, load, age of exhaust etc [28]. In the present study, the animals were exposed to aged resuspended diesel engine exhaust particles that was collected in a bag-filter during operation of a heavy duty fork-lift, i.e. NIST SRM 2975. Most previous studies employed freshly generated diesel exhaust, also including gases, however from a range of different vehicle engines [4,8,9]. These different diesel exhaust sources and methods may result in very different physico-chemical exposures. First, the role of volatile species can only be tested by direct exposure to fresh diesel engine exhausts and aged DEP may contain much lower concentrations of semivolatile compounds than achieved by direct exposure due to thermal, chemical and light-induced degradation. Second, the particle size distribution of resuspended DEP is coarser than the DEP emitted directly from a diesel engine. Here we observe a concentration maximum at approximately 145 nm and a mass median diameter of approximately 240 nm. When measured in exhaust directly from a diesel engine, diesel soot particles form both primary particles of up to 40 nm in size and the more abundant agglomeration (accumulation mode) particles. The fresh DEP soot mode is generally below 100 nm in seize, and a peak number in concentration of the carbonaceous soot occurs at approximately 60 nm [29,30]. Heavy duty diesel engines may show coarser size distributions than smaller engines. Kittelson et al. reported geometric volume mean diameters of around 125 nm for accumulation mode DEP particles [31]. Such differences in size may result in different deposition and translocation efficiencies as well as different toxicological mechanisms. Third, the chemical composition of the pure DEP will vary as function of trace metals in the fuel, additives, and lubricating oils. These factors may result in a highly abundant formation of inorganic particles in DEP exhaust [30,32] which is observed in ambient air [33]. Engine wear and corrosion can also affect the composition of the DEP exhaust dramatically [34]. Current analysis of the chemistry and water solubility of elements in SRM 2975 has shown that the total concentration of inorganic elements were in the order of only 0.3 wt%. Approximately 50 wt% of the elements were water soluble and dominated by Na, Mg, K, Ca, Si, P and Zn [Jensen, KA, in preparation]. The individual concentrations of other water-soluble well-known toxic trace elements were <30 ng/mg. The concentration of the 12 (phenanthrene to indeno(1,2,3-cd)pyrene) of the 16 US-EPA target PAHs were also relatively low making a total of 75.8 ± 6.3 ng/mg [35,36]. For comparison, a typical driving cycle for heavy duty diesel engines results in approximately 40 wt% carbonaceous soot, 13 wt% ash and other, 14 wt% sulphate and water, as well as 7 and 25 wt% unburned fuel and oil, respectively [31]. Docekal et al. [37] report 0.5 to 4 wt% in exhaust from a new diesel engine for passenger cars depending on engine load. It is evident that such differences in the exposure type offer a highly plausible explanation for the incongruent findings, i.e. different atmospheres exert different effects. It is crucial that these parameters are well-characterized in future DEP exposure studies to enable a future understanding of causal relationships between exposure and effects.

Besides the potential exposure, several other factors influence pup growth, alterations in growth may also be caused by e.g. changes in milk palatability, reduced milk availability, maternal behavior, or poor sucking ability in the pup [38]. Cross fostering and/or observation of interactions between the dam and her pups may help elucidate whether the observed effect is in fact an indirect (maternal) effect or a direct consequence of the DEP exposure.

The genotoxic effects of particles can be particle- or inflammation driven due to either the chemical composition, surface properties of the particles themselves, or induction of an inflammatory response. These mechanisms may generate reactive oxygen and nitrogen species with genotoxic effects as the result [39]. Both genotoxic and inflammatory effects of prenatal DEP exposure were pursued in the present study. DNA damage was evaluated by means of the comet assay, which is a sensitive and reliable method for detection of DNA strand breaks [40]. Besides directly induced DNA strand breaks, also breaks that are transiently present during repair of DNA lesions such as oxidative DNA damage, will be detected. DNA damage was furthermore evaluated by mRNA expression of *HO-1*, which is expressed during oxidative stress, as well as *OGG1 *and *ERCC1*, which are genes expressed during DNA repair. Elevated expression levels of *HO-1*, *OGG1*, and *ERCC1 *have previously been demonstrated in adult rats and mice after DEP exposure, together with an increased level of DNA strand breaks when evaluated by the comet assay [41,42]. DNA damage was not detected by the comet assay or by mRNA gene expression of defense genes in the present study, suggesting that DNA damage was not induced in the DEP exposed offspring. Prenatal exposure to DEP has previously been associated with genotoxic effects. An increased level of DNA deletions was observed in the offspring of mice orally exposed on day 10.5 to 15.5 of gestation to various doses of DEP (generated by a light-duty, four-cylinder passenger car) [10]. However, the transplacental exposure did not cause oxidative DNA damage in the embryo tissue, which is consistent with the observed results in the present study. Low induction of DNA-damage is expected from the relatively low concentration of PAHs and transition metals in SRM 2975 [36]. SRM 2975 also induce modest amounts of OH radicals (316 ± 148 nmol OH radical/mg) as compared to a tail-pipe deposit sample from a VW-1600 GTD (2,133 ± 1,393 nmol OH radical/mg) [Jensen, KA, in preparation]. Hence, the effects may be related to the carbonaceous core of DEP rather than the chemical compounds in this DEP sample. The inflammatory response in offspring exposed to DEP during fetal life was assessed by analyzing mRNA expression of the inflammatory cytokines *IL-6*, *MCP-1*, and *MIP-2*. Although insignificantly, the mean mRNA expression levels of inflammatory cytokines were slightly increased for these cytokines, indicating a tendency of inflammation. Increased mRNA expression levels of inflammatory cytokines have previously been observed in placentas of pregnant mice exposed to diesel exhaust by inhalation at similar concentrations [5]. In this study, the mRNA expression level of *IL-6 *was elevated approximately 10-fold. Thus, in utero exposure to DEP may be associated with increased inflammation in the offspring as well.

Results from several in vivo studies indicate that diesel exhaust exerts hormone like activity [4,7,8,11-13]. Because hormonally mediated events play a central role in the development and function of the central nervous system, it is possible that cognitive effects may arise from developmental exposure to endocrine disruptors [43]. When cognitive function was tested in the Morris water maze, control and exposed offspring performed similarly in learning and memory tasks, but DEP exposed female offspring showed enhanced function during the first trial of the spatial reversal learning task. A similar, although statistically insignificant pattern was observed in male DEP offspring. Prenatal events may under some circumstances facilitate cognition [43]. Thus, prenatal stress improved performance on the reversal task in the Morris water maze [44], a study of perinatal exposure to dieldrin reported facilitated retention of learning on a symmetrical maze [45], and prenatal dioxin exposure has been associated with enhanced cognitive function in several studies [46-48]. However, in the latter studies it was also observed that improvement of cognitive function was specific to the 8-arm radial maze [48]. Further, the dioxin exposed rats showed deficits in a non-spatial learning task [47]. It is therefore interesting that the only other published study of cognitive function after prenatal exposure to diesel exhaust reported reduced performance in the passive avoidance learning test in both male and female mice [49], indicating that also prenatal DEP may affect domains of cognitive function differentially.

## Conclusion

In utero exposure to NIST SRM 2975 DEP affected weight gain during lactation. Cognitive function and levels of inflammatory and genotoxic responses were generally similar in exposed and control offspring. The particle size and chemical composition of the DEP and differences in exposure method may play a significant role on the biological effects observed. Of specific interest would be a comparative study of the effects of prenatal exposure to fresh exhaust versus resuspended particles.

## Methods

### Animals

40 time-mated, nulliparous, young adult mice (C57BL/6BomTac, Taconic Europe, Ejby, Denmark) were supplied at GD 3. The animals were, upon arrival, randomly distributed in groups of five and housed in white polypropylene (type III) cages with bedding (Lignocel S8) and enrichment (soft paper wool and small aspen wood blocks, Lillico), under controlled environmental conditions (12 hour light-dark cycles with light starting at 6.00 a.m., temperature 21 ± 2°C, humidity 50 ± 5%, ventilation 13 air changes per hour). Food (Altromin 1324) and tap water were provided ad libitum. The day after arrival, i.e. GD 4, the animals were weighed and assigned to two groups of 20 animals each, with similar weight distributions.

The animal welfare committee, appointed by the Danish Ministry of Justice, granted ethical permission for the studies. All procedures were carried out in compliance with the EC Directive 86/609/EEC and with the Danish law regulating experiments on animals (permission 2006/561-1123).

### Exposure

The diesel exhaust particles were Standard Reference Material 2975 from the National Institute of Standards and Technology (NIST, Gaithersburg, MD, USA). The specific surface area was 90 mg^2^/g, as determined by multipoint (Brunauer, Emmett, and Teller) nitrogen adsorption (Micromeritics, Gemini 2375), corresponding well to the reported specific area of 90 mg^2^/g, reported by NIST. Pycnometric particle density was 2.1 g/cm^3^, measured by an AccuPyc 1330 (Micromeritics). Detailed chemical characterization has recently been conducted by e.g., [35] and [Jensen, KA, in preparation].

Exposure to DEP was performed in an 18 L inhalation chamber with walls of glass and stainless steel. The airflow in the chamber was dynamic with a flow of 20 L/min and the exposure atmosphere was evenly distributed, with a slightly negative inside pressure. The particles were delivered with a microfeeder and were aerosolized with a dispersion nozzle at a pressure of 5 bar (Frauenhofer Institute für Toxicologie und Aerosolforschung, Germany).

The two groups of mice were exposed to either filtered clean air or approximately 20 mg DEP/m^3 ^on GDs 7–19 for one hour/day. The dose level and exposure period were chosen as not to induce marked maternal toxicity or pup mortality based on published studies of developmental toxicity of diesel exhaust [4,5,7]. Results from previous studies in our laboratory confirm that this mode of DEP exposure is associated with detectable effects in adult mice [42,50,51]. In observation hereof and to minimize the use of pregnant animals, in addition to the concurrent control group, non-DEP positive control exposures of pregnant mice were not performed.

Exposure took place between 7.30 am and 2.30 p.m. During exposure, the animals were placed singly in the rooms of a cylindrical wire mesh cage (diameter 29 cm, height 9 cm) with radical partitions, holding ten mice at a time ("ten-room-pie"). The females were observed during exposure for signs of toxicity and returned to their cages less than 10 minutes after the end of the one hour exposure period. Body weight was recorded on GD 4, and before exposure on GD 5, 7, 10, 14, 18, and 19.

### Exposure Monitoring

The DEP mass concentration was controlled by filter sampling and adjusted to maintain an exposure of ~20 mg/m^3 ^total suspended particles. Particle samples were collected at 2 L/min using Millipore cassettes mounted with Millipore Fluoropore filter (φ = 2.5 cm; pore size = 0.45 μm). Each sample was collected for 4 minutes, and weighed using a Sartorius Microscale (Type M3P 000V001) [42,50]. Before sampling, the filters were equilibrated in an air-conditioned weighing room at 50%RH and 20°C and pre-weighed. After exposure, the gravimetric data were controlled after at least 24 hours re-equilibration in the air-conditioned weighing room.

The fine particle exposure (< 600 nm) was monitored using a GRIMM Sequential (Stepping) Mobility Particle Sizer (SMPS) consisting of a Long Electrostatic Classifier (Model No. 5.521; Serial No. 5LP 10209) connected to a GRIMM Condensation Particle Counter (Model 5.400). The SMPS data sampling and calculations were completed using the GRIMM software 5.477/02 v. 1.34 and operated in the fast scan mode, which allows a full size distribution analysis from 9.8 to 874.8 nm within 3 min and 38 sec. Data were corrected for both Classifier and CPC efficiency using the available software options. Particles were neutralized using a 3.7 MBq Am-241 source (Model No. 5.521). To avoid particle overloading in the SMPS, two impactors with nominal d_50 _cut-points of 1,185 and 805 nm were mounted externally and internally at the DMA inlet and thoroughly cleaned after each round of exposure. At the measured DEP density (2.1 g/cm^3^), the lower impactor stage has a d_50 _at 532 nm. Therefore SMPS data for D_p _> 500 nm were excluded in the subsequent data analysis. Similarly particle sizes below the D_p _midpoint 12.8 nm midpoint were excluded owing to excess electrical noise during fast scan operation. Coarse particle exposure (0.75 to > 15 μm) was measured using a GRIMM Dust Monitor (Model 1.105) at a resolution of 6 sec. The Dust Monitor particle sizes were subsequently recalculated to geometric means assuming an upper channel cut-point at 20 μm.

### Parturition and lactation

After termination of exposure on GD 19, the females were housed alone and were monitored for birth each day, early in the light cycle. The expected day of delivery, GD 20, was designated PND 0 for the pups. Weights of dams and individual pups were recorded on PND 2 and the pups were counted and sexed. On this day one pup with median body weight was removed from litters with at least 5 pups, and the lungs and liver were dissected, weighed, and snap frozen in cryotubes (NUNC) in liquid N_2_. Pup weights were also recorded on PND 9 and at weaning on PND 22. At weaning, one male and one female with median body weights from each litter were kept for behavioral testing and housed in groups of 5 to 6 mice of the same gender and prenatal exposure status. Thus, the same male and female from each litter took part in all behavioral testing. One male from each litter was allocated to other studies. The females who had not given birth, the dams, and remaining offspring were anaesthetized with a mixture of 1.5 mg/kg hypnorm (Jannsen) and 1.5 mg/kg dormico (Roche) and sacrificed by exanguination (withdrawal of heart blood) on PND 23 or 24. Blood from dams and offspring was stabilized in 72 μL respectively 30 μL 0.17 mol/L K_2_EDTA and kept on ice until centrifugation (2200 RCF at 4°C for 5 min) within 60 min of collection. The number of uterine implantation sites was determined in the dams, and the thymus, lungs, liver, and spleen were dissected, weighed, and snap frozen in cryotubes (NUNC) in liquid N_2_. All samples were stored at -80°C until analysis.

### mRNA expression

Total RNA was purified from liver tissue from two day old mouse pups and DNAse treated by SV Total RNA Isolation System (Promega) as described by the manufacturer. cDNA was synthesized from the RNA using TaqMan^® ^Reverse Transcription Reagents (Applied Biosystems) as described by the manufacturer. mRNA levels of *IL-6, MCP-1, MIP-2, HO-1, OGG1*, and *ERCC1 *were quantified by real-time RT-PCR with 18S as a reference gene. For *IL-6, MCP-1*, and *18S *RNA TaqMan predeveloped reaction kits (Applied Biosystems) were applied. For *MIP-2, HO-1, OGG1*, and *ERCC1 *probes and primers were designed as described previously for *MIP-2 *[52] and for *HO-1, OGG1*, and *ERCC1 *[53]. The final concentrations of these primers and probes were optimized to concentrations between 100 and 400 nm. mRNA levels of different genes were quantified in separate wells. In all assays, TaqMan^® ^Universal PCR Master Mix from Applied Biosystems was used. Each sample was run in triplicate on the ABI Prism 7300 Sequence Detector System. The target genes were normalized to 18S rRNA by subtracting the threshold cycle (Ct) for 18S (C_t, reference_) from the Ct value of the target gene (C_t, target_): ΔC_t _= C_t, target _- C_t, reference_. The relative expression of the target gene was calculated as 2^-ΔCt^. The standard deviation of the triplicates was below 15%. Positive and negative mRNA controls were included in each run in the mRNA measurements and the assays were validated to be linear over a 100 fold dilution. Repeated measurements of the same sample (the control) in separate experiments were not allowed to differentiate with more than 30% from the average. If the 18S content fell outside the range in which the PCR had been found to be quantitative, the measurements were excluded.

### The Comet assay

DNA strand breaks in liver of pups sacrificed on PND 2 were determined by the Comet assay as previously described in [54] with minor modifications [55]. Briefly, prepared liver cells are embedded in agarose on a glass microscope slide and lyzed to remove proteins and lipids. During alkaline electrophoresis, pieces of DNA migrate away from the nucleus towards the anode to produce a shape similar to a comet. After staining, the DNA remnants are visualized by fluorescent microscopy. As the smallest DNA strands move fastest, it is possible to evaluate the degree of DNA damage. The level of DNA damage was evaluated by measurements of tail length in 50 randomly selected nuclei from each subject. H_2_O_2 _exposed and unexposed A549 cells were included as an internal control of the comet assay.

### Hormone analysis

Total T_4 _concentrations were analyzed in plasma from dams and offspring at weaning using a modified Delfia T_4 _Time-resolved fluoroimmunoassay (cat.no. 1244-030, Perkin Elmer, Wallac Oy, Turku, Finland). Instead of the T_4 _standards and the T_4 _antibody supplied in the Delfia T_4 _kit, T_4 _standards in T_4_-free rat serum (30041), as well as biotinylated T_4 _antibody (30039) from Biovian Ltd., Finland were used. The assay was run as outlined in the protocol supplied by Biovian Ltd. using streptavidin microtitration strips 8 × 12 wells (4009-0010) and T_4 _assay buffer (1244-111) from Perkin Elmer.

### Postweaning growth and behavioral testing

All investigations were performed during the animals' light cycle, i.e. between 8.00 a.m. and 4.30 p.m. From PND 3, the experimenter was kept unaware as to which group an individual mouse belonged. The same observer was used in any one test. Exposed and control animals were tested alternately. Female and male animals were tested during different weeks. After weaning, the pups were weighed once every month until termination of behavioral tests (19 weeks).

#### Learning and memory (Morris water maze)

One female and one male per litter were tested. Females were tested at 12 and 16 weeks of age, males at age 13 and 17 weeks as described earlier [44] with minor modifications. The maze consisted of a circular white plastic pool, with a diameter of 100 cm and a height of 45 cm. The pool was surrounded by many external cues (wall lights, wall decorations of geometric figures in black plastic foil etc.), which were visible from within the pool and could be used by the mouse for spatial orientation. The pool was filled to a depth of 27 cm with water at room temperature. Four points on the rim of the pool, N, E, S and W (not true magnetic directions), were used as starting points and divided the pool into four arbitrary quadrants. A circular transparent platform (diameter 10 cm) was situated on a solid support and submerged 1 cm below the water surface, and thus invisible from water level.

The animals were tested in four daily trials using the four starting points assigned in a pseudo-random sequence. One standard-cage with 5–6 animals was placed near the pool. In each trial, the mouse was gently placed in the water facing the wall at the designated starting position. When the mouse swam to and climbed onto the platform, the trial was completed. If the animal failed to locate the platform within 60 sec, it was led to the platform. All animals were left to sit on the platform for 15 sec, before it was returned to the cage. The latency to reach the platform was measured by stopwatch.

The following scheme was used:

#### Learning

With the platform situated at the center of SW quadrant, the animals were trained until a stable performance was established, i.e. 4 trials for 5 consecutive days.

#### Memory

Three weeks after the learning period, the animals were tested again with the platform still in the SW quadrant of the pool. The animals were given 4 trials on each of 3 consecutive days.

#### New platform position I (reversal learning)

The day after the memory test, the animals were tested in a reversal procedure with the platform placed opposite the original location, i.e. in the center of the NE quadrant. The animals were tested for 4 trials.

#### New platform position II (new learning)

The following day, the platform was moved to the center of the pool, and the animals were tested for 4 trials.

#### Activity (open field)

One female and one male per litter were observed for a 3-min period in circular open field, i.e. the white pool used for learning and memory. The females were tested at the age of 14 weeks and the males at the age of 15 weeks. The mice started each trial in the center of the field, and the location of the animal in the field was registered in real-time with a device developed in our laboratory using contrast-sensitive computer controlled video tracking [56]. The number defecations in the 3-minute observation period were recorded manually. The video-tracking device calculated the total distance the animal moved.

### Statistics

The accepted level of significance was 0.05 and the litter was considered the statistical unit. Post-weaning, one male and one female per litter were evaluated under each test condition and the resulting data analyzed separately. Gestational parameters, mRNA levels of *IL-6, MCP-1, MIP-2*, and data from the Open field test were analyzed by the Mann-Whitney *U*-test. The remaining data were analyzed by analyses of variance (ANOVA), when relevant with repeated measures in trials or days (ANOVA). Analyses of co-variance (ANCOVA) were used to test the differences between the groups in lactational weight gain, birth weights, and pre-weaning pup weights while controlling for litter size. ANCOVA was also applied for the post hoc analyses for this endpoint. Analyses were performed in SYSTAT Software Package version 9 and MINITAB 14.

## Competing interests

The author(s) declare that they have no competing interests.

## Authors' contributions

KSH was substantially involved in the design of the study, acquisition and analysis of gestational and behavioral data, the statistical analyses, the interpretation of the results, and drafted the manuscript. KAJ made substantial contribution to the set-up of the exposure chamber, particle analysis, drafting of the manuscript regarding exposure characterization discussion of data, and revised the manuscript critically. PN carried out the molecular and comet analyses, and was involved in the drafting of the manuscript regarding these endpoints. CT carried out the analysis for T_4 _and the drafting of the manuscript regarding this endpoint. UV contributed substantially to the study design and interpretation of gene expression and comet data, drafting of the manuscript regarding these endpoints, and revised the manuscript critically. ATS was substantially involved in the acquisition and analysis of molecular and comet data, and revised the manuscript critically. HW conceived the study, set up the particulate exposure and the exposure protocol, and revised the manuscript critically. All authors have read and approved the final manuscript.
